# Parent-perceived recurrent pain in children: associations with maternal pain, depressiveness, socioeconomic status, and children's behavioural difficulties

**DOI:** 10.3389/fped.2024.1287343

**Published:** 2024-02-06

**Authors:** Laura Petri, Tanja Poulain, Mandy Vogel, Christof Meigen, Wieland Kiess, Andreas Hiemisch

**Affiliations:** ^1^ LIFE Child—Leipzig Research Center for Civilization Diseases, Leipzig University, Leipzig, Germany; ^2^ Department of Women and Child Health, Hospital for Children and Adolescents and Center for Pediatric Research (CPL), Leipzig University, Leipzig, Germany

**Keywords:** recurrent pain, maternal pain, maternal depressiveness, children’s behavioural difficulties, socioeconomic status, perceived pain

## Abstract

**Objectives:**

The current study aimed to examine the potential transgenerational associations between maternal pain and depressiveness and childhood pain, and to explore the associations between the children's difficulties and recurrent pain (defined as pain occurring at least once a month in the previous 6 month) in healthy children aged 3–13 years.

**Methods:**

We collected Data between 2015 and 2019 as part of the LIFE Child study in Germany and investigated associations of maternal pain and depressiveness, child age, sex, pubertal stage, emotional difficulties, conduct difficulties, hyperactivity/inattention, peer group difficulties, and prosocial skills, and family socioeconomic status with the frequency of parent-perceived headache, backache, and stomachache in a sample of 1,850 children (4,819 documented visits) using logistic and ordinal regression analyses.

**Results:**

Overall, 10.4%, 24.4%, and 45.2% of parents reported their children had recurrent backache, headache, and stomachache, respectively, with 5.5% of children were reported to experience all three types of pain simultaneously. Higher age, female sex, puberty, emotional difficulties, low family socioeconomic status, as well as higher maternal impairment due to pain and maternal depressiveness were significantly associated with more frequent pain.

**Conclusions:**

Our study suggests that maternal pain, maternal depressiveness, and lower family socioeconomic status as well as child's emotional difficulties are significantly associated with a higher frequency of recurrent pain in children perceived by their parents.

## 1 Introduction

Recurrent pain is pain that reoccurs over time. It is a common health issue in childhood (1) that significantly impacts the health and well-being not only of the child but also of their families (2). Recurrent pain is associated with lower quality of life (3), reduced school attendance (4), decreased social participation (5) and compromised emotional functioning (6) in children. Although recurrent pain in children is not bound to a specific diagnosis (7), it can manifest as chronic pain, i.e., pain that persists or recurs for at least three months (8). Prevalence data for children and adolescents varies due to differences in definitions, pain assessments, and populations and was reported to range from 8% to 83% for headache, 4% to 53% for stomachache, and 14% to 24% for backache (1). According to the nationwide “German Health Interview and Examination Survey for Children and Adolescents” (KiGGS) in 2003–2006 (9), the 3-month prevalence of pain recurring at least once per month was 64.5% among children aged 3–10 years, while 9.9% even reported pain at least once per week.

According to Bronfenbrenner's bio-ecological model, child development is influenced by individual differences and several environmental factors acting in different systems, namely the microsystem (interpersonal relationships), the mesosystem (inter-relations between microsystems), the exosystem (e.g., a family's socio-economic position), the macrosystem (conventions in a society), and the chronosystem (development over time) (10). This theory can be applied to various areas of development, including children's perception of pain. Potential risk factors are controversially discussed in the literature. On the individual level, the experience of pain may be influenced by sociodemographic and behavioural parameters. For example, previous studies indicate that girls experience higher levels of pain than boys (11, 12). Also, higher age and an early onset of puberty have been associated with more frequent pain, especially in girls (13, 14). Regarding behavioural characteristics, recurrent pain was shown to be associated with emotional or psychological distress (15). Here, the causal direction might be bidirectional: Emotional difficulties may lead to increased sensitivity to pain (16) while recurrent pain may also lead to emotional difficulties, such as sadness and depression (15). Further, it has been observed that peer group difficulties, such as bullying, are related to recurrent pain (17). On the microsystem level, family stressors, including depressive symptoms in a parent, might affect children's experience of pain (18, 19). It has been observed that children of parents who experienced recurrent pain are more likely to develop pain themselves (20–22). Psychosocial theories suggest that mothers can transmit pain to their children (23–25). Besides genetic and epigenetic factors, family socialization also play a crucial role in shaping how to cope with pain (22). With regard to the exosystem, lower family socioeconomic status has been associated with children's poorer physical and mental health (26, 27), which in turn might increase the risk of recurrent pain (28, 29). Socioeconomic status influences several factors that can lead to recurrent pain in the context of biopsychosocial health, including the environment in which a child grows up, the family, kindergarten and school, as well as other environmental factors such as safety and well-being (27, 30). As other aspects of child development, the perception of pain changes throughout development (chronosystem) and may be subject to social norms (macrosystem).

While previous research often investigated recurrent pain in older children and adolescents (31, 32), the present study assessed recurrent pain and related factors in a sample of younger children and (young) adolescents aged 3–13 years. As the early experience of pain may influence later development (33, 34), it is important to explore which factors relate to pain in early development. Here, we explore individual (child age, sex, and puberty status, but also behavioural difficulties) familial (maternal pain and depressiveness), and socio-economic factors (socio-economic status). Based on previous research finding, we expect that girls, older children, children in puberty, children with mothers experiencing pain or depressiveness, and children with lower socioeconomic status experience higher levels of recurrent pain.

## 2 Methods

The analyses are based on data collected within the LIFE Child study, a longitudinal prospective study conducted in Leipzig, Germany. Since 2011, this study has been recruiting infants, children, and adolescents from preschools, schools, or public health centres, among others, to investigate determinants of healthy development as well as risk factors for common diseases. Follow-up visits are conducted every year. All parents gave written informed consent before their children were included in the study. The study was designed in accordance with the Declaration of Helsinki and approved by the Ethics Committee of the Medical Faculty of the University of Leipzig (Reg. No. 477/19-ek). The LIFE Child study has been described in more detail elsewhere (35, 36). Children aged 3.0–13.5 years who participated at least once in the LIFE Child study between January 2015 and December 2019 and for whom information on pain, socioeconomic status, age, and sex was available were eligible for the current analysis. A total of 1,850 children (862 (46.59%) girls and 988 (53.41%) boys) met these criteria. 1,266 of these children (68%) had participated at more than one time point, and 888 (48%) had participated more frequently than twice. The number of participants and the number of observations for each of the used questionnaires is shown in the Supplementary Table S1.

## 3 Measures

All questionnaires used in the present study were completed by children's parents on a computer screen during the visit at the LIFE Child study center.

### 3.1 Child characteristics

#### 3.1.1 Pain

The frequency of parent-perceived pain among children was assessed using the parent-report form of a short version of the Gießen Complaints Form (GBB-KJ) (37, 38). It assesses the frequency of eight subjective health complaints within the last six months. The frequency is rated on a 5-point Likert scale (0 = rarely or never, 1 = once a month, 2 = almost every week, 3 = several times per week, 4 = daily). In our analysis, three pain items were considered, namely headache, stomachache, and backache. The GBB-KJ has been validated in 4- to 18-year-old children (37, 38).

### 3.1.2 Behavioural strengths and difficulties

The parent-report version of the Strengths and Difficulties Questionnaire (39, 40) was used to assess children's behavioural difficulties. The 25 items of this screening instrument comprise the following five scales: emotional difficulties, conduct difficulties, hyperactivity/inattention, peer group difficulties, and prosocial skills. Scores on each scale range from 0 to 10, with higher scores indicating more child's difficulties or—in the case of prosocial skills—child's strengths (41). The SDQ has been validated several times for children aged 4 years and older (39), but was already used for children aged as young as three years (42). In our study, cronbach's alpha, a marker for internal consistency/reliability, was 0.65 for emotional difficulties, 0.62 for hyperactivity/inattention, 0.56 for conduct difficulties, 0.63 for peer group difficulties, and 0.62 for prosocial skills, which represents an intermediate internal reliability.

#### 3.1.3 Puberty

Puberty stages were assessed by trained study assistants based on Tanner's Sexual Maturity Rating (SMR). SMR is an objective classification system used to document and track the development and sequence of secondary sexual characteristics in children during puberty (43). Tanner stage 1 was defined as prepubertal, while Tanner stage >1 was defined as pubertal (44).

### 3.2 Family characteristics

#### 3.2.1 Depressiveness

The mental health condition of participants' mothers was assessed using the Patient Health Questionnaire (45). The questionnaire was completed by mothers themselves. It is based on the diagnostic criteria in the Diagnostic and Statistical Manual of Mental Disorders (DSM-IV) (46). We analysed the depressiveness scale, which comprises 9 items answered on a 4-point Likert scale (0–3 level of intensity) (47). Final scores range from 0 to 27, with higher scores indicating more depressive symptoms in the past four weeks. Cronbach's alpha in the present study for the PHQ-9 questionnaire was 0.84 which represents a good internal reliability.

#### 3.2.2 Pain

Mothers' level of impairment by headache, stomachache, and backache (i.e., the same pain symptoms as assessed in children) in the past two weeks was assessed using the validated somatoform disorder scale of the Patient Health Questionnaire (45). The questionnaire was completed by mothers themselves. Each item was answered on a 3-point Likert scale, with higher scores indicate more impairment by pain (48).

#### 3.2.3 SES

The family's socioeconomic status (SES) was assessed using a parent-reported questionnaire including questions on their education, occupation, and equivalised disposable income. Information on these three parameters is combined to a composite score ranging from 3 to 21, with higher scores indicating higher SES (49, 50). We followed the categorization proposed by the KiGGS study (51) and divided our observations into three groups: low SES (3–9 points), middle SES (10–15 points), and high SES (16–21 points).

## 4 Statistical analyses

All statistical analyses were performed using R version 4.1.2 (52). In a first step, to assess the associations of child characteristics and family characteristics with child's pain (dependent variable as ordinal scale), ordinal regression analyses with one predictor (unadjusted models) were performed using the cumulative link mixed model (53), referenced as Model 1, in Tables 1, 2. In addition, to determine which predictor explained the variation in child's pain best, we used Nakagawa's R-squared to assess the proportion of variance explained by the independent variables (Supplementary Table S8) (54). In a second step, all characteristics that showed significant associations in the ordinal analyses (unadjusted models) were included in a multivariate ordinal regression analysis. One multivariate analysis was performed for child characteristics and another for family characteristics, referenced as Model 2, in Tables 3, 4. For all analyses, we also employed logistic regression models. In our logistic regression analyses we dichotomized the variable for each of the three domains of pain, and chose pain occurring at least once per month, i.e., response categories 1–4, as the threshold. Logistic regression results with one predictor (unadjusted models) are presented in the Supplements as Model 3 in Supplementary Tables S2, S3 and multivariate logistic regression results are presented in the Supplements as Model 4 in Supplementary Tables S4, S5. We additionally tested for interactions with the child's sex and age in unadjusted models, see in the Supplementary Tables S6, S7. All models were adjusted for multiple measurements per subject and multiple subjects per family by including a nested random effect. Results are presented as odds ratios, including the 95% confidence interval (CI).

**Table 1:** Model 1 ordinal analyses (unadjusted models): associations between child pain and child characteristics (3–13-year-olds, in 2015–2019, observations = 4,819). OR = Odds ratio (+95% confidence interval), controlled for multiple visits, and family relationships within the sample (as random effect).

Predictors↓		Headache	Stomachache	Backache
Age	OR (+ 95% CI) *p*	1.45 (1.39–1.51) <.001	0.94 (0.92–0.97) <.001	1.51 (1.43 −1.60) <.001
Sex (female)	OR (+ 95% CI) *p*	1.56 (1.19–2.04) 0.001	2.25 (1.85–2.73) <.001	1.35 (0.82−2.24) 0.238
Puberty stage (Tanner >1)	OR (+ 95% CI) *p*	4.65 (3.65–5.92) <.001	1.05 (0.85–1.30) 0.659	4.27 (2.81–6.48) <.001
Emotional difficulties	OR (+ 95% CI) *p*	1.62 (1.53–1.70) <.001	1.51 (1.44–1.57) <.001	1.37 (1.26–1.50) <.001
Hyperactivity/inattention	OR (+ 95% CI) *p*	1.07 (1.02–1.12) 0.003	1.04 (1.00–1.08) 0.016	1.03 (0.95–1.12) 0.449
Prosocial skills	OR (+ 95% CI) *p*	0.91 (0.88–0.94) <.001	0.94 (0.89–0.98) 0.009	0.92 (0.83–1.01) 0.105
Peer group difficulties	OR (+ 95% CI) *p*	1.21 (1.14–1.29) <.001	1.08 (1.02–1.14) 0.004	1.25 (1.12–1.39) <.001
Conduct difficulties	OR (+ 95% CI) *p*	1.17 (1.09–1.25) <.001	1.17 (1.11–1.23) <.001	1.09 (0.97–1.22) 0.128

**Table 2:** Model 1 ordinal analyses (unadjusted models): associations between child pain and family characteristics (3–13-year-olds, in 2015–2019, observations = 4,819). OR = Odds ratio (+ 95% Confidence interval), controlled for sex, age, multiple visits, and family relationships within the sample (as random effect).

Predictors ↓		Headache	Stomachache	Backache
Socioeconomic status (middle)	OR_low_ (+ 95% CI) *P* OR_high_ (+ 95% CI) *p*	1.72 (1.15–2.58) 0.008 0.77 (0.61–0.98) 0.032	0.97 (0.68–1.37) 0.850 0.95 (0.79–1.15) 0.600	1.08 (0.57–2.07) 0.807 0.54 (0.35–0.83) 0.004
Maternal Backache	OR (+ 95% CI) *p*	1.53 (1.27–1.84) <.001	1.35 (1.15–1.59) <.001	2.12 (1.57–2.86) <.001
Maternal Headache	OR (+ 95% CI) *p*	1.85 (1.51–2.25) <.001	1.86 (1.56–2.22) <.001	1.80 (1.31–2.47) <.001
Maternal Stomachache	OR (+ 95% CI) *p*	1.56 (1.21–1.99) <.001	1.80 (1.44–2.24) <.001	1.37 (0.92–2.03) 0.118
Maternal Depressiveness	OR (+ 95% CI) *p*	1.13 (1.09–1.16) <.001	1.06 (1.03–1.09) <.001	1.13 (1.08–1.19) <.001

**Table 3:** Model 2 ordinal multivariate analyses: associations between pain and child characteristics (3–13-year-olds, in 2015–2019, observations = 4,819). OR = Odds ratio (+ 95% Confidence interval), controlled for multiple visits, and family relationships within the sample (as random effect).

Predictors ↓		Headache	Stomachache	Backache
Age	OR (+ 95% CI) *p*	1.44 (1.36– 1.53) <.001	0.91 (0.89–0.94) <.001	1.50 (1.38–1.64) <.001
Sex (female)	OR (+ 95% CI) *p*	1.42 (1.09–1.84) 0.009	2.14 (1.76–2.59) <.001	–
Puberty stage (Tanner >1)	OR (+ 95% CI) *p*	1.14 (0.84–1.55) 0.398	–	1.08 (0.70–1.66) 0.732
Emotional difficulties	OR (+ 95% CI) *p*	1.54 (1.45–1.64) <.001	1.52 (1.45–1.60) <.001	1.32 (1.22–1.43) <.001
Hyperactivity/ inattention	OR (+ 95% CI) *p*	1.04 (0.98–1.09) 0.176	0.97 (0.93–1.01) 0.117	–
Prosocial skills	OR (+ 95% CI) *p*	0.99 (0.87–1.01) 0.957	0.96 (0.91–1.01) 0.174	–
Peer group difficulties	OR (+ 95% CI) *p*	0.94 (0.87–1.01) 0.112	0.94 (0.88–0.99) 0.031	0.99 (0.89–1.10) 0.883
Conduct difficulties	OR (+ 95% CI) *p*	1.06 (0.98–1.14) 0.172	1.06 (1.00–1.12) 0.047	–

**Table 4:** Model 2 ordinal multivariate analyses: associations between pain and family characteristics (3–13-year-olds, in 2015–2019, observations = 4,819). OR = Odds ratio (+ 95% Confidence interval), controlled for sex, age, multiple visits, and family relationships within the sample (as random effect).

Predictors ↓		Headache	Stomachache	Backache
Families’ Socioeconomic Status (middle)	OR_low_ (+ 95% CI) *p* OR_high_ (+ 95% CI) p	1.15 (0.71–1.88) 0.529 0.91 (0.67–1.22) 0.570	0.88 (0.56–1.38) 0.584 0.87 (0.67–1.13) 0.287	1.40 (0.79–2.48) 0.244 0.64 (0.43–0.97) 0.036
Maternal Backache	OR (+ 95% CI) *p*	1.11 (0.90–1.37) 0.312	1.02 (0.85–1.23) 0.838	1.57 (1.20–2.06) <.001
Maternal Headache	OR (+ 95% CI) *p*	1.69 (1.35–2.10) <.001	1.46 (1.20–1.77) <.001	1.34 (1.01–1.78) 0.042
Maternal Stomachache	OR (+ 95% CI) *p*	1.21 (0.96–1.60) 0.105	1.60 (1.27–2.01) <.001	–
Maternal Depressiveness	OR (+ 95% CI) *p*	1.06 (1.02–1.11) 0.006	1.07 (1.03–1.12) <.001	1.09 (1.04–1.15) <.001

## 5 Results

**Figure 1:**
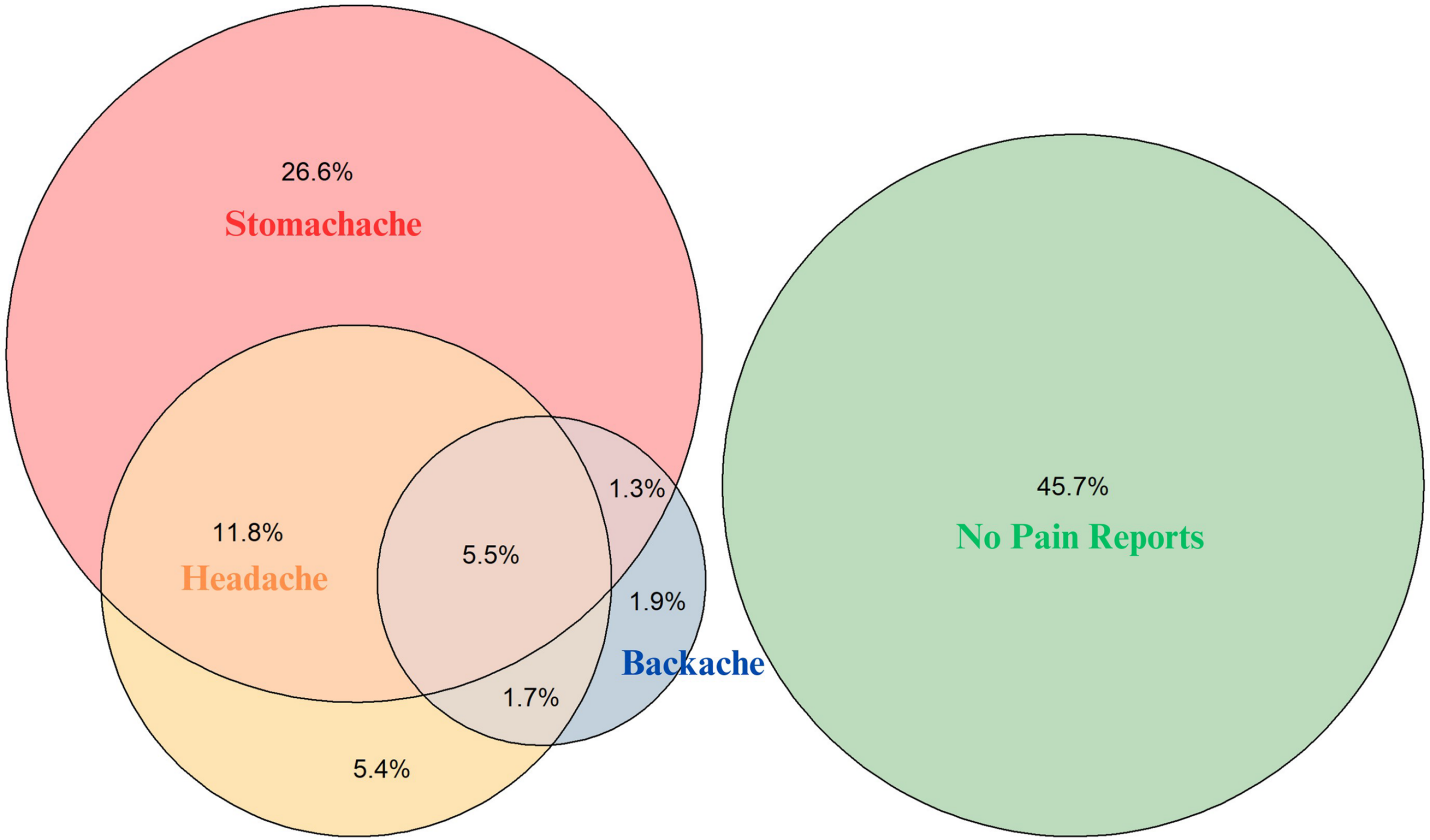
Overlap of different types of recurrent pain in 3- to 13-year-old children (*n* = 1,850).

The following descriptive results are based on information from the participants' first study visit (*n* = 1,850). The mean age was 7.0 (SD = 3.3). Most children (38.2%) were 3–4 years old. The distribution of sex was 988 (53.4%) male to 862 (46.6%) female. Regarding SES, children from families with low SES were underrepresented (7.7%), while children from families with high SES were overrepresented (38.7%). With respect to pain, recurrent headache was reported for 25.9% of girls and 23.2% of boys. Nearly half of the children (45.3%) were reported to have recurrent stomachache (52.8% of girls, 38.8% of boys), while recurrent backache was less common, reported for only 11.5% of girls and 9.4% of boys. In total, 54.3% of all children were reported to have recurrent pain in at least one domain. 4.4% of the children were reported to have recurrent pain in all three domains simultaneously. While a relatively large group of children (26.6%) were reported to only have recurrent stomachache (in the absence of recurrent headache or backache), very few children were reported to have only recurrent headache (5.4%) or only recurrent backache (1.9%), see Figure 1.

**Figure 2:**
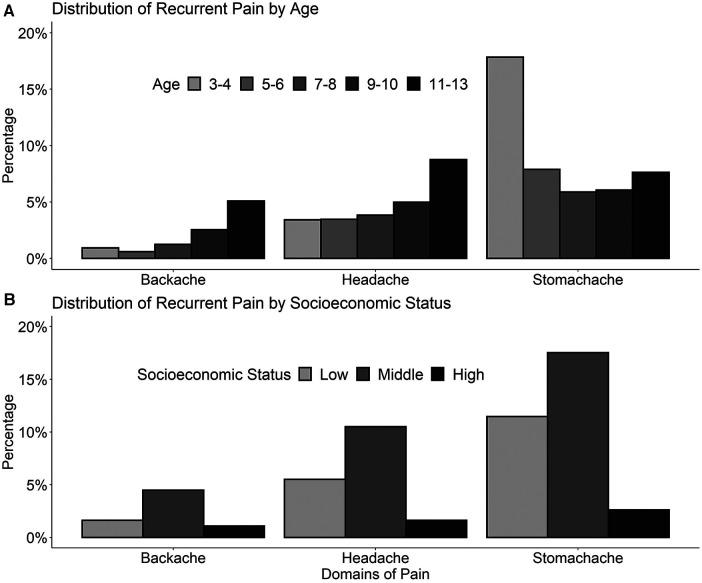
Frequency of recurrent stomachache, backache, and headache in 3- to-13-year-old children (*n* = 1,850) stratified by age (**A**), and socioeconomic status (**B**) recurrent pain was defined as pain at least once per month.

Figure 2 shows the distribution of recurrent pain by age group and SES. Backaches tended to increase with age, with a prevalence of under 1% in 3–4 and 5–6-year-olds, 2.5% in 9–10-year-olds, and 5.8% in 11–13-year-olds. Regarding headache, the prevalence increased with growing age, especially in 11- to 13-year-olds. In contrast to backache und headache, the prevalence of stomachache was especially high in 3- to 4-year-olds and decreased with growing age. Regarding SES, recurrent pain was reported more frequently in children from families with a middle SES.

The distributions of the other variables (puberty, behavioural difficulties, maternal depressiveness, and pain) are shown in Table 5.

**Table 5:** Characteristics of the study sample—first visit of the study.

	*N* = 1,850 *N* (%)	Female *n* = 862 *n* (%)	Male *n* = 988 *n* (%)
Age (years)			
3–4	707 (38.2%)	331 (38.4%)	376 (38.1%)
5–6	318 (17.2%)	153 (17.7%)	165 (16.7%)
7–8	249 (13.5%)	112 (13.0%)	137 (13.9%)
9–10	252 (13.6%)	106 (12.3%)	146 (14.8%)
11–13	324 (17.5%)	160 (18.6%)	164 (16.6%)
SES			
High	716 (38.7%)	319 (37.0%)	397 (40.2%)
Middle	992 (53.6%)	470 (54.5%)	522 (52.8%)
Low	142 (7.7%)	73 (8.5%)	69 (7.0%)
Headache			
0 rarely or never	1,398 (75.6%)	639 (74.1%)	759 (76.8%)
1 about once a month	326 (17.6%)	157 (18.2%)	169 (17.1%)
2 almost every week	85 (4.6%)	48 (5.6%)	37 (3.7%)
3 several times a week	29 (1.6%)	12 (1.4%)	17 (1.7%)
4 almost daily	12 (0.7%)	6 (0.7%)	6 (0.6%)
Backache			
0 rarely or never	1,658 (89.6%)	763 (88.5%)	895 (90.6%)
1 about once a month	133 (7.2%)	66 (7.7%)	67 (6.8%)
2 almost every week	39 (2.1%)	23 (2.7%)	16 (1.6%)
3 several times a week	10 (0.5%)	5 (0.6%)	5 (0.5%)
4 almost daily	10 (0.5%)	5 (0.6%)	5 (0.5%)
Stomachache			
0 rarely or never	1,012 (54.7%)	407 (47.2%)	605 (61.2%)
1 about once a month	584 (31.6%)	312 (36.2%)	272 (27.5%)
2 almost every week	153 (8.3%)	88 (10.2%)	65 (6.6%)
3 several times a week	77 (4.2%)	44 (5.1%)	33 (3.3%)
4 almost daily	24 (1.3%)	11 (1.3%)	13 (1.3%)
Puberty state			
1	1,291 (82.8%)	628 (78.6%)	663 (87.2%)
2	151 (9.7%)	82 (10.3%)	69 (9.1%)
3	68 (4.4%)	49 (6.13%)	19 (2.5%)
4	37 (2.4%)	29 (3.6%)	8 (1.1%)
5	12 (0.8%)	11 (1.4%)	1 (0.1%)
Child's difficulties	Mean (SD)	Mean (SD)	Mean (SD)
Emotional difficulties	1.77 (1.88)	1.85 (1.87)	1.71 (1.90)
Hyperactivity/inattention	3.67 (2.37)	3.39 (2.37)	3.91 (2.34)
Behavioural difficulties	2.08 (1.61)	1.96 (1.57)	2.19 (1.64)
Peer group difficulties	1.30 (1.53)	1.14 (1.36)	1.44 (1.66)
Prosocial skills	7.92 (1.66)	8.18 (1.56)	7.69 (1.71)
Maternal headache	*n* (%)	*n* (%)	*n* (%)
No impairment	418 (37.1%)	183 (35.1%)	235 (38.8%)
Slight impairment	586 (52.0%)	270 (51.7%)	316 (52.1%)
Severely impairment	124 (11.0%)	69 (13.2%)	55 (9.08%)
Maternal stomachache			
No impairment	847 (78.3%)	397 (80.5%)	450 (76.4%)
Slight impairment	208 (19.2%)	89 (18.1%)	119 (20.2%)
Severely impairment	27 (2.5%)	7 (1.4%)	20 (3.0%)
Maternal backache			
No impairment	477 (43.1%)	221 (43.2%)	256 (42.9%)
Slight impairment	480 (43.3%)	217 (42.5%)	263 (44.1%)
Severely impairment	151 (13.6%)	73 (14.3%)	78 (13.1%)
Maternal depressiveness	Mean (SD)	Mean (SD)	Mean (SD)
Depressiveness	3.19 (3.90)	3.24 (3.99)	3.15 (3.82)

### 5.1 Recurrent pain in association with sex, age and puberty

The following analyses were performed in the whole sample, including all follow-up visits. Odds ratios (OR) report the strengths of the association between the independent variables and the frequency of recurrent pain. In ordinal analyses (Table 1, Model 1), regarding age, we found a significant negative association with stomachache [OR_stomachache _= 0.94 (0.92–0.97), *p* < .001] and significant positive associations with headache (OR_headache _= 1.45 (1.39–1.51 *p* < .001) and backache [OR_backache _= 1.51 (1.43–1.60) *p* < .001]. Compared to boys, girls were reported to have significantly more headache [OR_headache _= 1.56 (1.19–2.04) *p* < .001] and stomachache [OR_stomachache _= 2.25 (1.85–2.73) *p* < .001]. Children in puberty (Tanner >1) were reported to have headache and backache more frequently than prepubertal children (Tanner = 1) (OR_headache _= 4.65 (3.65–5.92), *p* < .001, OR_backache _= 4.27 (2.81–6.48), *p* < .001). The association between pubertal stage and headache was significantly stronger in girls than in boys [OR_IA _= 1.99 (1.23–3.22), *p* = 0.005], see Supplementary Table S6 for all interactions results.

### 5.2 Recurrent pain in association with child's difficulties

**Figure 3:**
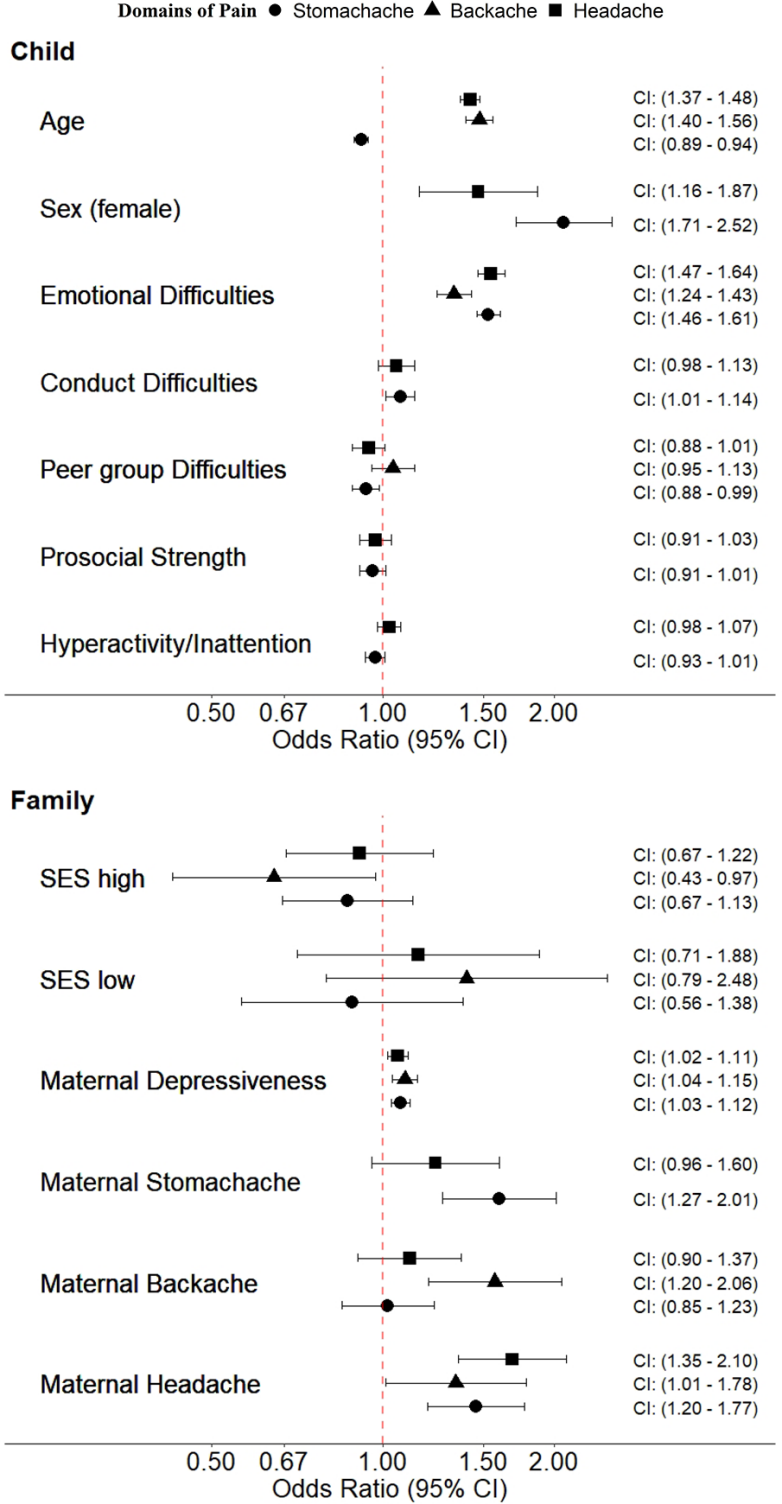
Associations between child and family-related characteristics with recurrent headache, stomachache, and backache. The figure visualizes the two multivariate ordinal models for the child's and family's characteristics with odds ratios including the 95% confidence intervals. Only characteristics that were significantly associated in the unadjusted models were included in the multivariate models.

Emotional and peer group difficulties were associated with a significantly increased frequency of pain reports in all three domains headache, stomachache, and backache (Table 1 Model 1). The association between backache and emotional difficulties was weaker in girls than boys [OR_IA _= 0.84 (0.70–0.99), *p* = 0.040], and became more pronounced as children's age increased [OR_IA _= 1.03 (1.00–1.05), *p* = 0.021] (Supplementary Table S6). Regarding symptoms of hyperactivity/inattention, conduct difficulties and poorer prosocial skills were associated with a significantly higher frequency of headache and stomachache (Table 1 Model 1). The results of the multivariate ordinal regression (Table 3 and Table 4 Model 2) analyses on the associations between pain and child characteristics are presented in Figure 3 (upper panel). As can be seen, associations with age, sex, and emotional difficulties remained significant, while associations with other child's difficulties did not.

### 5.3 Maternal pain and depressiveness in association with recurrent pain in children

Mothers' reports of their own pain impairment were associated with more frequent pain in their children in all three domains, e.g., maternal backache [OR_backache _= 2.12 (1.57–2.86), *p* < .001], or maternal headache [OR_headache _= 1.85 (1.51–2.25), *p* < .001] (Table 2 Model 1). The association between mothers' and children's backaches became more pronounced as children's age increased, e.g., OR_IA _= 1.13 (1.03–1.24), *p* = 0.011 (see Supplementary Table S7). Maternal depressive symptoms were associated with pain in all three domains e.g., headache OR_headache_ = 1.13 (1.09–1.16), *p* < .001 (Table 2 Model 1). The association between maternal depressiveness and stomachache in children was stronger in girls than boys: OR_IA_ = 1.06 (1.01–1.11), *p* = 0.021. Compared to children from families with middle SES, children from high SES families reported back pain significantly less frequently, while children from low SES families reported headache significantly more frequently (e.g., OR_headache_/SES_low _= 1.72 (1.15–2.58), *p* = 0.008 and OR_backache_/SES_high _= 0.54 (0.35–0.83), *p* = 0.004), (see Table 2 Model 1). In multivariate ordinal analyses, most associations, especially those between pain and maternal depressive symptoms and maternal pain in the same domain, remained significant (see Figure 3 and Tables 3, 4). In general, the results of the logistic models confirm those of the ordinal analyses.

## 6 Discussion

In line with other studies (1, 4, 9, 55) our results indicate a high prevalence of recurrent pain among 3–13-year-old children growing up in an urban environment in Germany. Importantly, we found a higher prevalence of pain in childhood than the representative German KiGGS study (55). For example, in our study, the prevalence of recurrent stomachache and recurrent headaches in girls were 45.2% and 24.4%, respectively, compared to 33.7% and 20.1% in the KIGGS study. One possible explanation for these differences is that our sample was more homogeneous and basically included children from urban regions only.

In line with the bio-ecological approach of Bronfenbrenner, the findings of the present study suggest that children's experience of pain, as one facet of child development, is shaped by several individual and environmental factors. Regarding individual factors, our findings indicate that pain frequency increases with age and the onset of puberty, which is consistent with previous research (1, 4, 13, 14, 56). In contrast to another previous study, this association was more pronounced in girls than in boys (57). Potential explanations of sex differences concern hormones, as oestrogen can affect pain sensitivity and perception (58). Further, there are sex differences in pain processing, which may at least partly be socio-culturally driven, in the sense that showing or admitting to be in pain is more accepted in girls than boys (59). It is important to note that differences in frequency between sexes persist into adulthood, so there may be other contributing factors (60). However, we also found that the association between back pain and emotional difficulties was stronger in boys than in girls. This may be an indication that boys are more likely than girls to have back pain when they have emotional difficulties. As expected (61, 62), emotional difficulties were an important dependent variable associated with pain, which may be considered as the internalizing character of recurrent pain (63).

Regarding family characteristics (microsystem), our findings suggest that maternal health is also associated with pain in children. If a child's mother reported more depressive symptoms, the frequency of recurrent pain in all three domains increased. In contrast, in a meta-analysis from 2020, the non-significant pooled correlation coefficient suggested that there was no consistent association between parental depression and pain frequency in children (64). In addition, our analyses revealed a significant association between a mother's pain and her child's pain of the same type. This correlation was also observed for all three domains of recurrent pain in the current study, even in multivariate analyses. Previous studies analysing the association between parents with recurrent pain and their children's risk of reporting recurrent pain found that these children are more likely to have recurrent pain than children whose parents do not have recurrent pain (2, 31, 65, 66) and our results generally confirm this association.

On the exosystem level, we showed that lower socioeconomic status was associated with more backache and headache. This association could be due to fewer resources available in terms of education and financial or emotional assets, which could lead to lower health awareness or pain coping strategies (28–30, 67).

In summary, our findings suggest that maternal health, including impairment by pain and depressive symptoms, is associated with more frequent pain in children. These transgenerational correlations show that it is necessary to consider pain and depressiveness in mothers to understand recurrent pain in childhood. Furthermore, our results suggest that emotional and conduct difficulties, peer group interactions, and pubertal changes are key factors associated with the frequency of recurrent pain in children.

### 6.1 Strength and limitations

One critical limitation of the study is the lack of investigation of paternal pain/ depressiveness in contrast to maternal pain/ depressiveness, caused by the small observation size of fathers participating in the LIFE Child study. Also, the observation is relatively homogeneous considering the socioeconomic status, e.g., low SES families were underrepresented in our study, which may limit the generalisability of the findings. Our mothers' reports of their children's pain may have been influenced by their own pain impairment. Further, there is the potential of recall bias, as depressed mothers could be more likely to report higher frequency of pain in their children than others. Also, the potential influence of drug therapy on maternal factors was not examined. Another limitation of this study is that we did not include social factors, such as quality of life, among the factors influencing the development of recurrent pain in childhood. Additionally, we did not consider school attendance frequencies or other school-specific variables among school-aged older children. While the questionnaire Gießen Complaints Form (GBB-KJ) and the Strengths and Difficulties Questionnaire (SDQ), are formally validated for children aged 4 and older, it is worth noting that in our study, we also administered the questionnaire to children as young as 3 years. Finally, we performed cross-sectional analysis. Therefore, assumptions on causality are not possible.

The strengths of the study include its large observation size, controlling for multiple visits and family relationships within the observation, the investigation of interactions with sex and age and the distinction between three specific domains of pain.

## 7 Conclusions

In conclusion, this study provides insight into several factors associated with parent-perceived recurrent pain in children. Child characteristics such as age, sex, and emotional difficulties were significantly associated with pain frequency in several domains. In addition, family characteristics such as lower socioeconomic status, maternal pain and maternal depressive symptoms were also associated with increased pain frequency in children. The finding that a specific type of pain (e.g., back pain) in the mother was associated with the same type of pain in the child (i.e., back pain) provides additional support for the existence of a transgenerational correlation between pain of the mother and pain of the child. The findings underline the importance of a multi-dimensional assessment of pain, considering both child and family factors.

## Data Availability

Data cannot be shared publicly because of ethical restrictions. Publishing data sets is not included in the informed consent provided by the study participants. Furthermore, the data protection concept of LIFE requests that all (external as well as internal) researchers interested in accessing the data sign a project agreement. Researchers who are interested in accessing and analysing data collected in the LIFE Child study may contact the data use and access committee dm@life.uni-leipzig.de.
