# Supplementary material for: Parent-perceived recurrent pain in children: associations with maternal pain, depressiveness, socioeconomic status, and children's behavioural difficulties
Source: Front Pediatr. 2024 Feb 6;12:1287343. doi: 10.3389/fped.2024.1287343 (PMC10876899; doi:10.3389/fped.2024.1287343)
Supplement: Supplementary file 1 [file Table1.pdf]

# *Supplementary Material*

## 1 Supplements Table 1

Table of measures: Number of observations and participants for each observed predictor in the analyses.

| **Predictors ↓** | **Number of Observations** | **Number of Participants** |
|---|---|---|
| Child’s Pain | 4819 | 1850 |
| Family’s Socioeconomic Status | 4819 | 1850 |
| Child’s Strengths and Difficulties | 4819 | 1832 |
| Maternal Depressiveness | 4819 | 1850 |
| Maternal Pain | 2747 | 1082 |
| Child’s Pubertal Stage | 4060 | 1559 |

## 2 Supplements Table 2

Model 3 Logistic analyses (unadjusted models): Associations between child pain and child characteristics (3–13-year-olds, in 2015 – 2019, Observations =4819). OR= Odds Ratio (+ 95% Confidence interval), controlled for multiple visits, and family relationships within the sample (as random effect).

| **Predictors ↓** | | **Headache** | **Stomachache** | **Backache** |
|---|---|---|---|---|
| Age | OR (+ 95% CI)<br>p | 1.49 (1.42 – 1.55)<br>< .001 | 0.95 (0.93 – 0.98)<br>< .001 | 1.58 (1.47 – 1.69)<br>< .001 |
| Sex (female) | OR (+ 95% CI)<br>p | 1.50 (1.15 – 1.96)<br>0.003 | 2.19 (1.82 – 2.63)<br>< .001 | 1.33 (0.73 – 2.41)<br>0.351 |
| Puberty Stage (Tanner > 1) | OR (+ 95% CI)<br>p | 6.10 (4.63 – 8.05)<br>< .001 | 1.10 (0.89 – 1.37)<br>0.382 | 4.75 (2.91 – 7.76)<br>< .001 |
| Emotional difficulties | OR (+ 95% CI)<br>p | 1.61 (1.52 – 1.71)<br>< .001 | 1.42 (1.36 – 1.49)<br>< .001 | 1.38 (1.24 – 1.53)<br>< .001 |
| Hyperactivity/ Inattention | OR (+ 95% CI)<br>p | 1.06 (1.01 - 1.11)<br>0.016 | 1.03 (1.00 – 1.07)<br>0.096 | 1.01 (0.93 – 1.11)<br>0.765 |
| Prosocial strength | OR (+ 95% CI)<br>p | 0.91 (0.85 – 0.97)<br>0.004 | 0.954 (0.91 – 1.00)<br>0.055 | 0.91 (0.81 – 1.02)<br>0.093 |
| Peer group difficulties | OR (+ 95% CI)<br>p | 1.22 (1.14 – 1.31)<br>< .001 | 1.07 (1.02 – 1.13)<br>0.011 | 1.23 (1.09 – 1.39)<br>< .001 |
| Conduct difficulties | OR (+ 95% CI)<br>p | 1.16 (1.08 – 1.24)<br>< .001 | 1.15 (1.09 – 1.21)<br>< .001 | 1.07 (0.94 – 1.22)<br>0.281 |

## 3 Supplements Table 3

Model 3 Logistic analyses (unadjusted models): Associations between child pain and family characteristics (3–13-year-olds, in 2015 – 2019, Observations =4819). OR= Odds Ratio (+ 95% Confidence interval), controlled for sex, age, multiple visits, and family relationships within the sample (as random effect).

| **Predictors ↓** | | **Headache** | **Stomachache** | **Backache** |
|---|---|---|---|---|
| Families' Socioeconomic Status (middle) | $OR_{low}$ (+ 95% CI) | 1.59 (1.04 – 2.45) | 1.01 (0.71 – 1.43) | 1.13 (0.54 – 2.37) |
| | p | 0.034 | 0.974 | 0.753 |
| | $OR_{high}$ (+ 95% CI) | 0.72 (0.56 – 0.92) | 0.92 (0.76 – 1.11) | 0.57 (0.34 – 0.93) |
| | p | 0.008 | 0.390 | 0.025 |
| Maternal Backache | OR (+ 95% CI) | 1.49 (1.22 – 1.81) | 1.30 (1.10 – 1.53) | 2.04 (1.43 – 2.93) |
| | p | < .001 | 0.001 | < .001 |
| Maternal Headache | OR (+ 95% CI) | 1.76 (1.43 – 2.17) | 1.78 (1.49 – 2.14) | 1.81 (1.24 – 2.65) |
| | p | < .001 | < .001 | 0.002 |
| Maternal Stomachache | OR (+ 95% CI) | 1.69 (1.29 – 2.21) | 1.80 (1.41 – 2.28) | 1.41 (1.40– 1.41) |
| | p | < .001 | < .001 | < .001 |
| Maternal Depressiveness | OR (+ 95% CI) | 1.12 (1.09 – 1.16) | 1.06 (1.03 – 1.09) | 1.13 (1.06 – 1.20) |
| | p | < .001 | < .001 | < .001 |

## 4 Supplements Table 4

Model 4 Logistic multivariate analyses: Associations between child pain and child characteristics (3–13-year-olds, in 2015 – 2019, Observations =4819). OR= Odds Ratio (+ 95% Confidence interval), controlled for multiple visits, and family relationships within the sample (as random effect).

| Predictors ↓ | | Headache | Stomachache | Backache |
|---|---|---|---|---|
| Age | OR (+ 95% CI)<br>p | 1.43 (1.35 – 1.52)<br>< .001 | 0.93 (0.90 – 0.96)<br>< .001 | 1.63 (1.62-1.63)<br>< .001 |
| Sex (female) | OR (+ 95% CI)<br>p | 1.32 (1.01– 1.73)<br>0.038 | 2.07 (1.72 – 2.50)<br>< .001 | - |
| Emotional Difficulties | OR (+ 95% CI)<br>p | 1.56 (1.46 – 1.67)<br>< .001 | 1.41 (1.34 – 1.48)<br>< .001 | 1.39 (1.38 – 1.40)<br>< .001 |
| Hyperactivity/ Inattention | OR (+ 95% CI)<br>p | 1.03 (0.97 – 1.09)<br>0.249 | - | - |
| Prosocial Strengths | OR (+ 95% CI)<br>p | 0.96 (0.89 – 1.04)<br>0.348 | - | - |
| Peer Group Difficulties | OR (+ 95% CI)<br>p | 0.91 (0.84 – 0.99)<br>0.035 | - | 1.02 (1.01 – 1.02)<br>< .001 |
| Conduct Difficulties | OR (+ 95% CI)<br>p | 1.06 (0.97 – 1.15)<br>0.159 | 1.06 (1.00 – 1.11)<br>0.035 | - |

## 5 Supplements Table 5

Model 4 Logistic multivariate analyses: Associations between child pain and family characteristics (3–13-year-olds, in 2015 – 2019, Observations =4819). OR= Odds Ratio (+ 95% Confidence interval), controlled for sex, age, multiple visits, and family relationships within the sample (as random effect).

| **Predictors ↓** | | **Headache** | **Stomachache** | **Backache** |
|---|---|---|---|---|
| Families' Socioeconomic Status (middle) | $OR_{low}$ (+ 95% CI) | 0.89 (0.53 – 1.51) | | |
| | p | 0.664 | | |
| | $OR_{high}$ (+ 95% CI) | 0.78 (0.57 – 1.07) | | |
| | p | 0.124 | | |
| Maternal Backache | OR (+ 95% CI) | 1.22 (0.95 – 1.57) | 1.04 (0.87 – 1.25) | 2.76 (1.69– 4.51) |
| | p | 0.461 | 0.629 | < .001 |
| Maternal Headache | OR (+ 95% CI) | 1.70 (1.31 – 2.20) | 1.49 (1.23 – 1.81) | 2.29 (1.35– 3.86) |
| | p | < .001 | < .001 | 0.001 |
| Maternal Stomachache | OR (+ 95% CI) | 1.47 (1.06 – 2.03) | 1.57 (1.22 – 2.01) | 1.23 (0.67 – 2.25) |
| | p | 0.009 | < .001 | 0.485 |
| Maternal Depressiveness | OR (+ 95% CI) | 1.06 (1.01 – 1.12) | 1.08 (1.04 – 1.12) | 1.06 (0.95– 1.17) |
| | p | 0.043 | < .001 | 0.239 |

## 6 Supplements Table 6

Model 1 ordinal analyses (unadjusted models) and interactions with sex (female) and age (3–13-year-olds, in 2015 – 2019, Observations =4819). OR= Odds Ratio (+ 95% Confidence interval), controlled for multiple visits, and family relationships within the sample (as random effect).

| **Predictors ↓** | | **Headache Interactions** | **Stomachache Interactions** | **Backache Interactions** |
|---|---|---|---|---|
| Age | Sex (female) | | | |
| | OR (+ 95% CI) | 1.11 (1.03 – 1.19) | 1.10 (1.04 – 1.16) | 1.07 (0.97 – 1.19) |
| | p | 0.004 | 0.001 | 0.187 |
| Puberty Stage (Tanner > 1) | Sex (female) | | | |
| | OR (+ 95% CI) | 1.99 (1.23 – 3.22) | 2.05 (1.33 – 3.19) | 1.48 (3.38 – 0.65) |
| | p | 0.005 | 0.001 | 0.356 |
| | Age | | | |
| | OR (+ 95% CI) | 0.78 (0.66 – 0.924) | 0.96 (0.827 – 1.13) | 0.91 (1.13 – 1.16) |
| | p | 0.004 | 0.689 | 0.438 |
| Emotional Difficulties | Sex (female) | | | |
| | OR (+ 95% CI) | 1.03 (0.93 – 1.13) | 0.98 (0.93 – 1.09) | 0.84 (0.70 – 0.99) |
| | p | 0.599 | 0.900 | 0.040 |
| | Age | | | |
| | OR (+ 95% CI) | 1.01 (0.99 – 1.03) | 1.00 (0.98 – 1.01) | 1.03 (1.00 – 1.05) |
| | p | 0.198 | 0.582 | 0.021 |
| Hyperactivity/ Inattention | Sex (female) | | | |
| | OR (+ 95% CI) | 0.97 (0.88 – 1.06) | 1.01 (0.94 – 1.08) | 0.96 (0.82 – 1.13) |
| | p | 0.460 | 0.875 | 0.619 |
| | Age | | | |
| | OR (+ 95% CI) | 1.01 (0.99 – 1.03) | 1.00 (0.99 – 1.01) | 1.01 (0.99 – 1.03) |
| | p | 1.121 | 0.913 | 0.558 |
| Prosocial Strengths | Sex (female) | | | |
| | OR (+ 95% CI) | 1.11 (0.98 – 1.25) | 0.99 (0.90 – 1.09) | 1.08 (0.88 – 1.32) |
| | p | 0.101 | 0.864 | 0.484 |
| | Age | | | |
| | OR (+ 95% CI) | 1.01 (0.99 - 1.03) | 1.00 (0.99 – 1.02) | 1.00 (0.97 – 1.03) |
| | p | 0.524 | 0.565 | 0.958 |
| Peer Group Difficulties | Sex (female) | | | |
| | OR (+ 95% CI) | 1.12 (0.99 – 1.28) | 0.96 (0.86 – 1.06) | 1.18 (0.86 – 1.46) |
| | p | 0.081 | 0.427 | 0.122 |
| | Age | | | |
| | OR (+ 95% CI) | | | |
| | p | 1.00 (0.98 – 1.02) | 1.00 (0.98 – 1.01) | 1.03 (1.00 – 1.06) |
| | | 0.761 | 0.737 | 0.072 |
| Conduct Difficulties | Sex (female) | | | |
| | OR (+ 95% CI) | 1.02 (0.90 – 1.16) | 1.01 (0.91 – 1.11) | 0.79 (0.63 – 0.99) |
| | p | 0.750 | 0.895 | 0.041 |
| | Age | | | |
| | OR (+ 95% CI) | 1.00 (0.98 – 1.02) | 1.00 (0.98 – 1.01) | 1.03 (0.99 – 1.06) |
| | p | 0.767 | 0.817 | 0.116 |

## 7 Supplements Table 7

Model 1 ordinal analyses (unadjusted models) and interactions with sex (female) and age (3–13-year-olds, in 2015 – 2019, Observations =4819). OR= Odds Ratio (+ 95% Confidence interval), controlled for sex, age, multiple visits, and family relationships within the sample (as random effect).

| **Predictors ↓** | | **Headache**<br>**Interactions** | **Stomachache**<br>**Interactions** | **Backache**<br>**Interactions** |
|---|---|---|---|---|
| Families' Socioeconomic Status (middle) | Sex (Female) | | | |
| | $OR_{low}$ (+ 95% CI) | 1.58 (0.67 – 3.72) | 1.42 (0.71 – 2.85) | 0.34 (0.08 – 1.49) |
| | p | 0.297 | 0.319 | 0.151 |
| | $OR_{high}$ (+ 95% CI) | 1.45 (0.896 – 2.34) | 1.23 (0.86 – 1.75) | 1.41 (0.52 – 3.85) |
| | p | 0.129 | 0.256 | 0.499 |
| | Age | | | |
| | $OR_{low}$ (+ 95% CI) | 1.09 (0.94 – 1.26) | 1.06 (0.95 – 1.18) | 1.39 (1.09 – 1.78) |
| | p | 0.244 | 0.310 | 0.008 |
| | $OR_{high}$ (+ 95% CI) | 1.10 (1.02 – 1.19) | 1.03 (0.97 – 1.09) | 1.03 (0.91 – 1.15) |
| | p | 0.013 | 0.331 | 0.678 |
| Maternal Backache | Sex (Female) | | | |
| | OR (+ 95% CI) | 0.94 (0.65-1.36) | 1.07 (0.78 - 1.47) | 0.96 (0.57 – 1.83) |
| | p | 0.733 | 0.673 | 0.951 |
| | Age | | | |
| | OR (+ 95% CI) | 0.96 (0.900-1.03) | 0.96 (0.91 – 1.02) | 1.13 (1.03 – 1.24) |
| | p | 0.228 | 0.174 | 0.011 |
| Maternal Headache | Sex (Female) | | | |
| | OR (+ 95% CI) | 1.00 (0.68-1.49) | 1.28 (0.91 – 1.79) | 1.00 (0.53 – 1.86) |
| | p | 0.983 | 0.153 | 0.988 |
| | Age | | | |
| | OR (+ 95% CI) | 1.04 (0.97-1.12) | 1.02 (0.95 – 1.06) | 1.07 (0.97 – 1.19) |
| | p | 0.229 | 0.837 | 0.151 |
| Maternal Stomachache | Sex (Female) | | | |
| | OR (+ 95% CI) | 0.77 (0.47-1.27) | 1.06 (0.69 – 1.62) | 0.59 (0.27 – 1.30) |
| | p | 0.313 | 0.793 | 0.193 |
| | Age | | | |
| | OR (+ 95% CI) | 0.96 (0.88-1.05) | 0.99 (0.92 – 1.06) | 1.01(0.88 – 1.13) |
| | p | 0.378 | 0.754 | 0.986 |
| Maternal Depressiveness | Sex (Female) | | | |
| | OR (+ 95% CI) | 1.02 (0.96-1.08) | 1.06 (1.01 – 1.11) | 1.09 (0.99 – 1.20) |
| | p | 0.599 | 0.021 | 0.07 |
| | Age | | | |
| | OR (+ 95% CI) | 1.00 (1.00-1.01) | 1.01 (1.00 – 1.01) | 1.01 (1.00 – 1.02) |
| | p | 0.315 | 0.071 | 0.102 |

# 8 Supplements Table 8

Extract of Pseudo-$R^2$ results

| Stomachache | Conditional Pseudo-$R^2$ |
| --- | --- |
| Emotional Difficulties | 0.454 |
| Maternal Depressiveness | 0.433 |
| Maternal Stomachache | 0.468 |

| Backache | Conditional Pseudo-$R^2$ |
| --- | --- |
| Emotional Difficulties | 0.868 |
| Maternal Depressiveness | 0.886 |
| Maternal Backache | 0.859 |

| Headache | Conditional Pseudo-$R^2$ |
| --- | --- |
| Emotional Difficulties | 0.558 |
| Maternal Depressiveness | 0.572 |
| Maternal Headache | 0.568 |
